# Retraction Note: A ketogenic diet for the management of binge-eating disorder: a pilot study

**DOI:** 10.1007/s40519-026-01916-7

**Published:** 2026-11-03

**Authors:** Yixuan Liang, Angela Taylor, Chuyu Qiu

**Affiliations:** 1 Department of Neuroscience, Johns Hopkins University, Baltimore, MD USA; 2 Department of Clinical Nutrition, Johns Hopkins University, Baltimore, MD USA; 3 Department of Molecular and Cellular Biology, Johns Hopkins University, Baltimore, MD USA

**Retraction Note: Eating and Weight Disorders - Studies on Anorexia, Bulimia and Obesity (2026) 31:42** **https://doi.org/10.1007/s40519-026-01843-7**

The Editor-in-Chief has retracted this article for legal and research integrity reasons.

Following publication, the publisher has been contacted by Dr. Verena Haas and Charité-Universitätsmedizin Berlin claiming incorrect information in the article.

Publisher contacted the authors regarding the raised concerns, however they were unable to provide a satisfactory explanation or documentation, including appropriate ethics approval document.

During investigation, it was determined that Dr. Verena Haas is not an author of the article. The false authorship information was added without her involvement or permission. The article also contained incorrect information on her institutional affiliation. Additionally, the article incorrectly stated the involvement of Charité-Universitätsmedizin Berlin in the study reported in the article.

The article in its published form has therefore been removed for legal reasons. The authorship information of the article has been updated.

None of the authors has responded to correspondence from the Publisher regarding this retraction notice.

